# Ultrafast charge separation dynamics in opaque, operational dye-sensitized solar cells revealed by femtosecond diffuse reflectance spectroscopy

**DOI:** 10.1038/srep24465

**Published:** 2016-04-20

**Authors:** Elham Ghadiri, Shaik M. Zakeeruddin, Anders Hagfeldt, Michael Grätzel, Jacques-E. Moser

**Affiliations:** 1 Photochemical Dynamics Group , Ecole Polytechnique Fédérale de Lausanne, CH-1015 Lausanne, Switzerland; 2 Lausanne Centre for Ultrafast Science (LACUS), Ecole Polytechnique Fédérale de Lausanne, CH-1015 Lausanne, Switzerland; 3 Laboratory for Photonics and Interfaces, Ecole Polytechnique Fédérale de Lausanne, CH-1015 Lausanne, Switzerland; 4 Laboratory of Photomolecular Science, Ecole Polytechnique Fédérale de Lausanne, CH-1015 Lausanne, Switzerland

## Abstract

Efficient dye-sensitized solar cells are based on highly diffusive mesoscopic layers that render these devices opaque and unsuitable for ultrafast transient absorption spectroscopy measurements in transmission mode. We developed a novel sub-200 femtosecond time-resolved diffuse reflectance spectroscopy scheme combined with potentiostatic control to study various solar cells in fully operational condition. We studied performance optimized devices based on liquid redox electrolytes and opaque TiO_2_ films, as well as other morphologies, such as TiO_2_ fibers and nanotubes. Charge injection from the Z907 dye in all TiO_2_ morphologies was observed to take place in the sub-200 fs time scale. The kinetics of electron-hole back recombination has features in the picosecond to nanosecond time scale. This observation is significantly different from what was reported in the literature where the electron-hole back recombination for transparent films of small particles is generally accepted to occur on a longer time scale of microseconds. The kinetics of the ultrafast electron injection remained unchanged for voltages between +500 mV and –690 mV, where the injection yield eventually drops steeply. The primary charge separation in Y123 organic dye based devices was clearly slower occurring in two picoseconds and no kinetic component on the shorter femtosecond time scale was recorded.

Dye-sensitized solar cells (DSCs) are promising candidates for solar energy conversion applications. These devices do not rely on rare or expensive materials, so they could be more cost-effective than cells based on silicon and thin-film technologies. Recently, DSCs device efficiency has reached a maximum power conversion efficiency of over 12% using donor- bridge - acceptor (D-π-A) zinc porphyrin dye in combination with a cobalt-based redox mediator^1^.

The performance of DSCs is based on kinetics competition between the electron injection from the sensitizer to an electron collecting material, usually TiO_2_, regeneration of the oxidized dye with redox electrolyte and unwanted back reactions of injected electrons recombining with oxidized dye molecules or oxidized species of redox electrolyte^2^. A challenge in this field is that the kinetics of charge carriers may be altered in complete devices showing top performances. A deep understanding of many parameters controlling the overall performance is crucial for achieving improvements in performance. Despite numerous studies, there is still a debate on the electron injection time scale for the optimized solar cells and according to the proposed “kinetics redundancy”, the optimized solar cells might not have ultrafast electron injection kinetics^3^. Existing studies are mainly based on the classical pump-probe transient absorption spectroscopy, which is widely used to measure the kinetics of electron injection processes. Since optical transparency is required, to perform transient absorption studies, only model systems based on a transparent TiO_2_ thin film sensitized with various dyes and semiconductors in different environments (solid samples or in solution) were investigated so far^3^,^4^,^5^,^6^,^7^. It should, however, be noted that the most efficient liquid-based solar cell devices are not transparent. Indeed, these devices are based on a double layer of TiO_2_ film, which contains a scattering layer made of 400 nm TiO_2_ particles deposited on top of a mesoporous transparent layer^8^. The resulting light transmittance of the cell is less than 15% in the visible region and, hence, conventional transient absorption spectroscopy in transmission mode cannot be applied in this case. Despite the importance of the subject, the kinetics of electron injection in actual optimized, opaque dye-sensitized solar cell devices under working conditions has not so far been reported.

We aim here to investigate the dynamics of charge carriers directly in fully functional devices, using potential control and state-of-the-art pump-probe diffuse reflectance spectroscopy. Despite the great potential of the latter technique, only a few studies can be found in literature investigating its implementation and application. We aim to demonstrate that diffuse reflectance spectroscopy is of great value for time-resolved analysis of photophysical processes in opaque or highly absorbing materials. Time-resolved diffuse reflectance spectroscopy was first reported by Wilkinson *et al.*^9^ in microsecond time regime in 1981 followed by Bowman *et al.*^10^ and Asahi *et al.*^11^,^12^. The technique was utilized on scattering systems like powders of organic microcrystals, and by Furube *et al.*^13^ on DSCs under open circuit condition. Here we have developed an ultrafast time-resolved pump-probe diffuse reflectance spectrometer with a sub-200 femtoseconds time-resolution. This required application of novel optical design for the collection of diffuse reflected light. In addition and for the first time, we combined the femtosecond time-resolved diffuse reflectance laser spectroscopy with potential control and photovoltage measurements.

Furthermore, the technique has enabled us to investigate the charge separation kinetics in DSCs based on photoanodes of other TiO_2_ film morphologies. For example, we studied samples of anodized nanotubes on Ti foil^14^,^15^ and nanostructured fibers^16^,^17^. These samples have exhibited promising behavior in cell performance but are not optically transparent and are not suitable for investigation with pump-probe transmission based transient absorption technique.

Our studies reveal that the charge separation dynamics in Ru-based dye in the complete device is ultrafast and is indeed affected by the morphology of the TiO_2_ film. We observed an early charge recombination in scattering TiO_2_ particles, TiO_2_ fibers and anodized TiO_2_ nanotubes. These recombinations had different amplitudes and were not previously reported for small particles and are rationalized in terms of different electron mobility and trapping states in different TiO_2_ films. Under an applied voltage bias condition from +500 mV up to –690 mV, the kinetics of the electron injection from the dye excited-state into the oxide remains ultrafast. However, the injection yield decreases at the bias point of –690 mV. In contrast to Ru-complex based dye, the organic D-π-A dye Y123 exhibited slower charge injection kinetics. The excited-state lifetime of Y123 dye is measured to be 50 ps. The time constant of the electron injection process is measured being 1.1 ps. While this classifies as ultrafast, it is about one order of magnitude slower than for Ru-based dyes, which was measured to have features in femtosecond time scale.

## Results and Discussions

**Figure 1:**
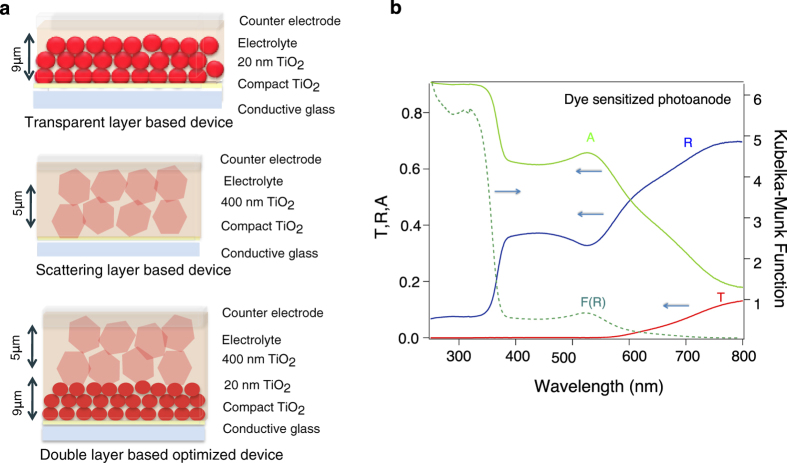
Device architecture and optical properties of complete photoanode. (**a**) Schematic of the optimized highly efficient liquid DSC based on single layer and double layer TiO_2_ films. This represents examples of the devices that have been studied. (**b**) Steady-state optical parameters of a Z907 sensitized TiO_2_ double layer photoanode applied in highly efficient DSC devices. Total transmittance (Red), diffuse reflectance (blue), absorptance (green) and Kubelka- Munk function (dashed green) are depicted. Kubelka-Munk function and absorptance are defined according to equations (1) and (3) depicted in the method section. The total transmittance of the cell in the visible and infrared region is less than 15%. The Kubelka-Munk function spectrum follows the shape of the absorptance spectrum.

Figure 1a shows the schematics of the standard optimized high-performance liquid solar cell. In the conventional DSC scheme, the mesoporous layer is made of 20 nm-diameter interconnected TiO_2_ particles. Although this structure offers a large surface area for dye adsorption, Rayleigh scattering with this size of TiO_2_ particles is small, resulting in high transparency of the dye-sensitized film in a broad spectral region. A significant amount of light (70% in the near infrared region) is transmitted without interacting with dye molecules in the cell. The working electrode applied in highly efficient devices is based on a TiO_2_ double layer film^18^, sensitized with dye molecules on top of a TiCl_4_-treated conductive glass. The structure of these samples is shown in Fig. 1a. The first layer is a transparent mesoporous anatase TiO_2_ film, consisting of interconnected spherical nanoparticles (20 nm). Another layer made of 400 nm-diameter TiO_2_ particles is deposited on top of the transparent layer. Figure 1b shows the total transmittance, total reflectance and total absorptance of the Z907 dye-sensitized TiO_2_ double layer film based DSC photoanode. The 400 nm particles act as light scattering centers enhancing light absorption by increasing the light pathway within the film. Consequently, the total transmittance of the cell in the visible and near-infrared region is less than 15% as it can be seen in Fig. 1b. This suggests that the diffuse reflectance spectroscopy is the only versatile optical laser spectroscopy technique capable of studying such devices. The Kubelka-Munk function, F(R) spectra is derived from diffuse reflectance of the film according to equation (3), presented in the method section. The F(R) spectrum is compared with the absorptance spectrum of the opaque photoanode in Fig. 1b. As it is seen, the Kubelka-Munk spectrum follows the shape of the absorptance curve, and the similarity in both spectra is observed. The peak around 520 nm corresponds to the Z907 dye ground state absorption that serves as an absorbing medium. The shoulder at 380 nm corresponds to the absorption of TiO_2_ substrate that serves as the scattering media in Kubelka-Munk theory.

Two types of liquid electrolyte-based devices were selected for the present study. The first type of DSC is prepared with a Ru-complex sensitizer (Z907) in combination with an iodide/ triiodide based redox electrolyte in 3-methoxypropionitrile solvent. This combination was reported to result in highly stable devices when subjected to light and thermal stress during long-term aging^19^,^20^. The second type of cell is based on the organic D-π-A, sensitizer Y123 and a cobalt complex-based redox electrolyte. This type of device yielded a power conversion efficiency of over 9%^21^,^22^ and over 12% in combination with a porphyrin dye^1^. The thickness of both TiO_2_ layers affects both photocurrent and photovoltage, which were optimized in earlier studies in terms of final power conversion efficiency^8^.

### Femtosecond diffuse reflectance spectroscopy on operational DSC device based on Z907 sensitizer

In order to unravel the electron injection dynamics in dye-sensitized opaque solar cells, we utilized pump-probe diffuse reflectance spectroscopy. We applied this technique to the study of Z907 dye-sensitized TiO_2_ films, which are from the same family of N719 and N3 Ru-based dyes. According to earlier transient absorption studies on N719 (cis-bis (isothiocyanate)bis(4,4′-dicarboxylic-2,2′-bipyridyl) ruthenium(II)) and N3 (cis-di(thiocyanate)bis(2,2′-bipyridyl-4,4′-dicarboxylic acid)ruthenium(II)) dye-sensitized TiO_2_ films, the transient absorption spectrum around 800 nm is assigned to the oxidized dye molecules^13^,^23^,^24^,^25^ and the absorption spectrum around 1200 nm is attributed to absorption by conduction band electrons^13^,^26^,^27^. The kinetics of electron injection in the complete device is studied by monitoring the evolution of oxidized dye molecules and photo-injected electrons in TiO_2_. The oxidized dye molecules are monitored at the characteristic absorption onset in the visible wavelength region at 670 nm or in the NIR region at 840 nm, and photo-injected electrons are monitored at 1200 nm.

**Figure 2:**
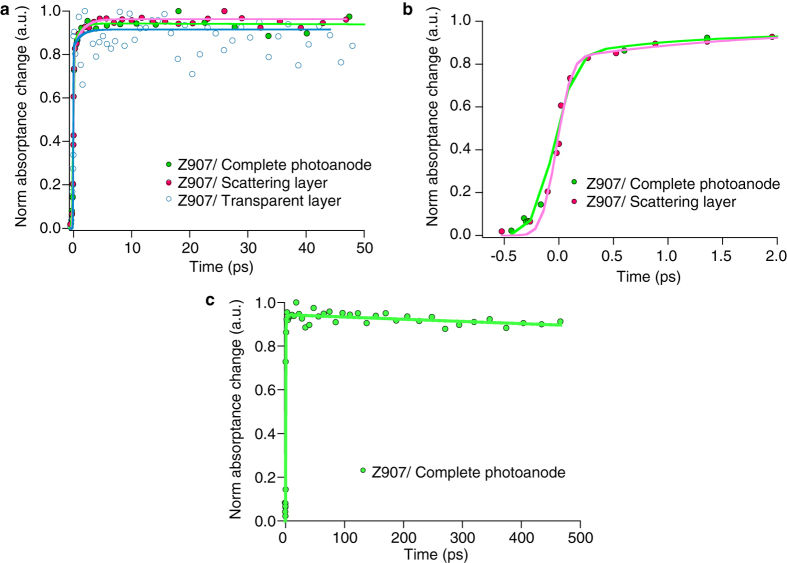
Transient diffuse reflectance of dye-sensitized TiO_2_ photoanodes. Samples are covered with MPN solvent. λ_ex_ = 600 nm and λ_obs_ = 850 nm. (**a**) Evolution of Z907 oxidized dye molecule anchored on transparent film (blue markers), scattering film (red marker) and double layer film (green marker) recorded up to 50 ps after excitation. Lines are fitted to convoluted exponential function. (**b**) Kinetics of oxidized dye molecule anchored on scattering film (red) and complete photoanode (green) up to 2 ps after excitation. Fitting parameters for complete photoanode are A = 0.11, τ_1_ = 1.08 ps, σ = 0.158 ps and μ = −0.05 ps. Fitting parameters for scattering layer are A = 0.15, τ_1_ = 1.75 ps, σ = 0.109 ps and μ = −0.024 ps. (**c**) Kinetics of oxidized dye molecule in full photoanode until 500 ps after excitation. Fitting parameters are A = 0.1, τ_1_ = 1.18 ps, σ = 0.164 ps, μ = −0.05 ps, B = 0.9 and τ_2_ = 4 ns. A and B are the pre-exponential factors and τ is the time constant. σ and μ are the broadening and zero onset of Gaussian function fit to cross-correlation of the pump and probe.

Figure 2a,b compare the early and later time evolution of absorptance of oxidized dye molecules anchored on three different TiO_2_ films in the presence of MPN solvent. The samples are excited at 600 nm. The time delayed diffuse reflected probe beam is measured at 840 nm. Transient absorptance change is extracted from the measured transient diffuse reflectance change given by equation (2), depicted in the method section. In Fig. 2a,b, it is seen that in the presence of MPN solvent, the kinetics of electron injection in double layer film resembles that of transparent film made of small TiO_2_ particles. All samples have an instrument response-limited transient absorptance onset within 200 fs and a slow rise of the signal with a time constant of 1.1 ps.

The first ultrafast response limited rise of the signal corresponds to the ultrafast electron injection from dye to TiO_2_ conduction band. Ultrafast fluorescence studies performed by Chergui and coworkers^28^ on N719 sensitized TiO_2_ small particles films revealed that the electron injection occurs with a time constant of 10 fs for non-thermalized levels of the dye and 120 fs from the thermalized level. The second slow rise component up to 5 ps is observed in all three TiO_2_ films was also previously reported in measurements of transparent films by different research groups^29^,^30^. Studies by Wenger *et al.*^29^ assigned this feature to the presence of dye aggregates in the films, which have a larger distance for electron injection and, therefore, less electronic coupling for electron transfer process. Sundström *et al.* proposed another description in terms of a two-state mechanism. They assigned the fast and slow components to the injection from the singlet and the triplet excited-states of the ruthenium complex, respectively^30^.

Figure 2c illustrates the long time kinetics of the evolution of oxidized dye molecules up to 500 ps after excitation in the working cell based on TiO_2_ double layer film. Remarkably, the kinetics features a decay component in several hundred picoseconds after excitation. The trace is fitted by an exponential decay function with a time constant of 4 ns. It should be noted that all measurements shown in Fig. 2 are performed at very low excitation intensities. For measurements shown in Fig. 2, the excitation energy of each pulse at the sample is 200 nJ. The repetition rate is 0.5 kHz. The beam diameter is close to 500 μm. Under these conditions, the excitation irradiance is 102 μJ cm^−2^. With having the repetition rate of 0.5 kHz, the excitation power is 51 mW cm^−2^, which is equivalent to 50% of the sun irradiance power at AM 1.5 condition. In addition, the number of photons normalized to the volume of TiO_2_ within the irradiation area is about 2.15 × 10^11^. This is significantly smaller than the number of adsorbed dye molecules on the microscopic surface of TiO_2_ film, within the irradiated area which is 9.8 × 10^16^ (assuming one monolayer of adsorbed dye molecules). Hence, our observations are not due to some non-linear effects.

**Figure 3:**
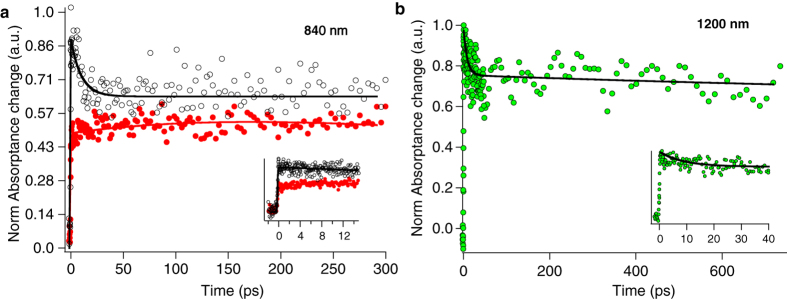
Transient absorptance on complete device. Normalized transient absorptance deduced from femtosecond diffuse reflectance measurements on a DSC composed of Z907 dye-sensitized TiO_2_ double layer. (**a**) Probed at 840 nm monitoring the kinetics of oxidized dye molecules in the presence of MPN solvent (red markers) and Iodide based electrolyte Z946 (black markers); the solid lines correspond the fit to the result by a two exponential function. The inset shows the short delay time scan of the observed kinetics at 840 nm in the presence of MPN solvent and electrolyte. (**b**) The observed kinetics in the vicinity of electrolyte measured at 1200 nm monitoring the kinetics of photo-injected electrons in TiO_2_. The solid lines correspond the fit to the result by exponential function. The time constant of exponential fit to the measurements at 840 nm is τ_1_ = 9.22 ps and for trace measured at 1200 nm is τ_1_ = 8.9 ps. The pump wavelength is 530 nm and pulse intensity is 200 nJ.

The excitation intensity dependence of the observed kinetics is also presented in Supplementary information, Figure S4. All traces measured at low excitation intensities can be fitted by a single exponential function. This early decay kinetics is again observed when the evolution of oxidized dye molecules is monitored in the visible wavelength region at 670 nm (Supplementary Figure S5). Moreover, the same depleting kinetics can be observed (Fig. 3b) when photo-injected electrons are monitored at 1200 nm in the presence of the electrolyte. Therefore, we assign the early decay kinetics observed for the complete opaque DSC in the presence of MPN solvent, to the early back recombination of photo-injected electrons with oxidized dye molecules.

**Figure 4:**
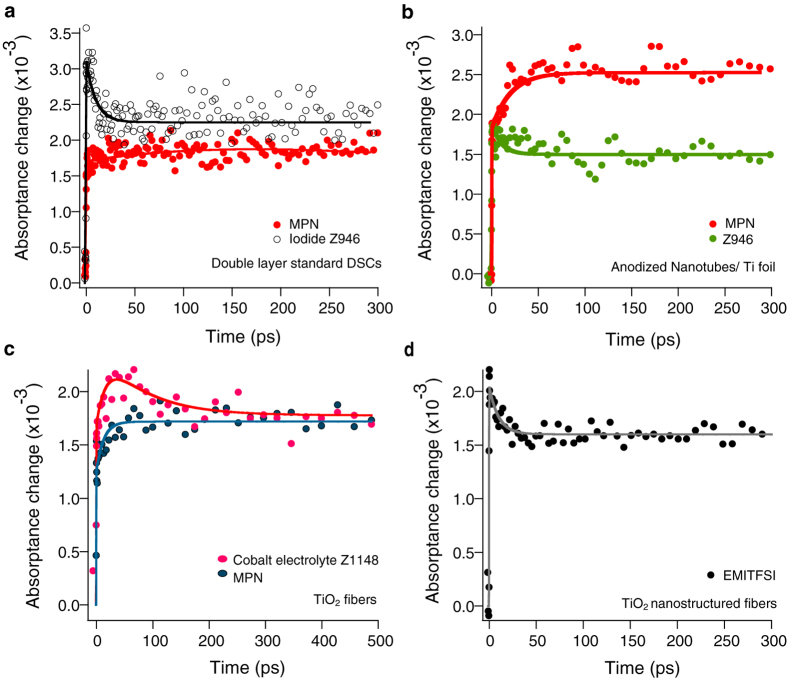
Transient absorptance on different nanostructured opaque TiO_2_ films based devices. Transient absorptance change deduced from transient diffuse reflectance measurements on DSCs based on different TiO_2_ opaque films. λ_ex_ = 530 nm and λ_obs_ = 840 nm. (**a**) Standard DSC based on TiO_2_ double layer dipped in MPN solvent (red) and iodide-based redox electrolyte (black). 30% of the signal decays in 20 ps in the vicinity of electrolyte. (**b**) DSC based on anodized TiO_2_ nanotubes on Ti foil dipped in MPN solvent (red) and iodide-based redox electrolyte (green). 16% of the signal decays in 20 ps after excitation in the vicinity of the electrolyte. (**c**) DSC based on TiO_2_ fibers in MPN solvent (blue) and cobalt-based redox electrolyte (pink). In the presence of cobalt electrolyte, 20% of signal decays in 200 ps after excitation. (**d**) DSC based on TiO_2_ fibers dipped in redox-inactive ionic liquid, EMITFSI (black), 28% of the signal decays in 20 ps after excitation. The measurements are performed at the same excitation intensities.

The kinetics of back recombination is even more strongly accelerated in the presence of the electrolyte. Traces in Fig. 3a compare the measurements in the presence (black markers) and absence of redox electrolyte (red markers) on double layer TiO_2_ film. In the vicinity of the redox electrolyte, the formation of the signal is again ultrafast, and the sub-200 fs ultrafast component is still present. As it can be observed, 26% of the signal of the oxidized dye molecule decays with a time constant of τ_1_ = 9 ps. The same fast decay kinetics are also present in the samples dipped in the redox-inactive ionic liquid, 3-methyl-1-ethylimidazolium bis (trifluoromethane) sulfonimide (EMITFSI), (see Fig. 4d). Therefore, the observed kinetics cannot be assigned to processes like reductive quenching of the excited-state of the dye molecules by redox electrolyte. The accelerated charge recombination in the presence of both redox active and redox inactive electrolyte in some picoseconds after excitation is rationalized by electric fields induced by the charges in the electrolyte and charge screening effects. The local electric field induced by ions present in the redox electrolyte or ionic liquid at the surface is accelerating the charge recombination between electron-hole geminate pair after initial charge separation.

Figure 3b provides more evidence, as photo-injected electrons in TiO_2_ are directly monitored at 1200 nm. The trace is fitted with two exponential decay functions. The rate constant of the fast decay component of photoelectrons measured at 1200 nm is 1.1 × 10^11^ s^−1^ and for the slower decay kinetic is 5.917 × 10^8^ s^−1^, giving a lifetime of 8.9 ps and 1.7 ns, respectively. Interestingly, these time constants are consistent with the kinetics fit values of measurements at 840 nm monitoring oxidized dye molecules. Taken together, the observed decay kinetics at 840 nm and the mirror kinetics at 1200 nm are due to an early back recombination of photo-injected electrons with the oxidized dye molecules anchored on the surface of TiO_2_ particles.

Therefore, a significant observation in our measurements of the DSC devices based on scattering particles and all other TiO_2_ morphologies (as it is discussed below), is that the kinetics of electron back recombination with oxidized dye molecule has features in the picosecond to nanosecond time scale. This observation is remarkably different to what is normally stated in the literature for model systems of transparent films made of small particles. The small particles based films are the only morphology that has been studied to date. In earlier studies of small TiO_2_ particles sensitized with Ru dyes based on transient absorption spectroscopy at visible or NIR region or by 2D-IR spectroscopy^23^,^31^,^32^,^33^, such electron back recombination was not observed and the oxidized dye molecule was accepted to be stable until much longer time scale of microseconds. We observe that the kinetics is accelerated in full device relative to what is so far accepted for small particles. In a complementary study, presented in Supplementary Table S1, the photovoltaic, optical and structural characteristics of the transparent and double layer based devices are depicted. The value of photocurrent normalized to the light absorptance of the scattering layer is about 30% less than that for transparent layer based device. This indicates that in big particles some fraction of photoelectrons are lost and is consistent with the early back recombination of electrons and oxidized dye molecules observed in laser spectroscopy measurements.

In addition, the pump-probe diffuse reflectance technique has also enabled us to investigate the electron injection profiles in many other DSC devices. These studies include measurements of different opaque nanostructured TiO_2_ films such as TiO_2_ nanofibers^16^ and TiO_2_ nanotubes. TiO_2_ nanotubes are prepared by anodization of Ti foil as the substrate^14^. Due to opacity, these samples could never be studied by transmission based transient absorption technique.

Figure 4 shows the dynamics of electron injection on Z907 dye-sensitized TiO_2_ films of different morphologies monitored at 840 nm. Panel a shows the kinetics of charge separation in Z907 dye-sensitized standard double layer based DSC device in the presence and absence of redox electrolyte. Panel b depicts the pump-probe diffuse reflectance measurements on Z907 dye-sensitized anodized TiO_2_ nanotube film on Ti foil. The same studies are performed on dye-sensitized TiO_2_ nanostructured fibers. Measurements of fibers in the presence of cobalt-based electrolyte (CO^II^/CO^III^) and also redox-inactive electrolyte (EMITFSI), are shown in panel c and d, respectively. It is interesting that in all measurements the electron injection is still in the ultrafast regime and recombination features with different amplitudes comparable to that of double layer film is present. In standard full DSC based on double layer film 30% of the signal decays in 20 ps after excitation, this compares to only 16% for DSCs based on anodized nanotubes.

The observed kinetics in the presence of redox-inactive ionic liquid suggests that the decay kinetics cannot be due to the reductive quenching of the dye excited-state by redox electrolyte. The observed difference in early back recombination of photoelectrons in the TiO_2_ films of nanoparticles and anodized nanotubes is rationalized in terms of morphological parameters of the two films such as trap state distribution.

We hypothesize that photo-injected electrons may get trapped in the TiO_2_ particle surface states where they form geminate pairs with holes (oxidized dye molecules) and result in the observed early fast recombination in different TiO_2_ morphologies with different amplitudes. It should be noted that, our control studies (Figure S3) shows that at low excitation intensities of 0.25 sun irradiance at AM1.5, the excited dyes inject more than 3.8 × 10^16^ cm^−3^ electrons into the TiO_2_, which is much smaller than the trap state density (10^18^ cm^−3^ to 10^20^ cm^−3^) reported for TiO_2_ films^14^. One should also consider the energetic distribution of these traps. For instance, the trap state distribution in the anodized nanotubes is measured using macroscopic techniques like charge extraction experiments by Hagfeldt *et al.*^14^. The nanotubular electrodes have a trap state distribution significantly different from nanoparticulate electrodes. Nanotubes possess relatively deeper traps with a characteristic energy of the exponential distribution more than twice than that of nanoparticulate electrodes. Throughout time-resolved terahertz studies, Schmuttenmaer *et al.*^34^ have claimed that the low mobility in polycrystalline TiO_2_ nanotubes is not only due to scattering from grain boundaries or disorders as is in other nanomaterials but instead results from a single sharp resonance from exciton-like trap states. These observations are in good agreement with our spectroscopy studies. Indeed, electrons can get more localized in energetically deeper traps in nanotubes films and, therefore, less early back recombination with oxidized dye molecule is observed in these films in comparison with nanoparticles based films.

Our observations show direct evidence of an ultrafast electron injection occurring in a complete Ru-dye based DSC device. This has not been confirmed so far for DSCs having all components like scattering layer, electrolyte, conductive glass, etc. Charge injection from the amphiphilic Ru^II^(bipyridyl) Z907 dye in all different TiO_2_ morphologies was observed to take place in the sub-200 fs time scale. We observed that the kinetics of charge separation is indeed influenced by the morphological parameters of the TiO_2_ substrate. The photo-injected electron in TiO_2_ fibers, nanotubes, and 400 nm particles shows prompt back recombination kinetics with oxidized dye molecules in the picoseconds-nanoseconds time scale after excitation. Electronic parameters like density of trap states and energetic of trapped electrons, i.e. how deep electrons are trapped, and consequently the mobility of electrons, might play a vital role in the behavior of photo-injected electrons after initial interfacial charge separation.

**Figure 5:**
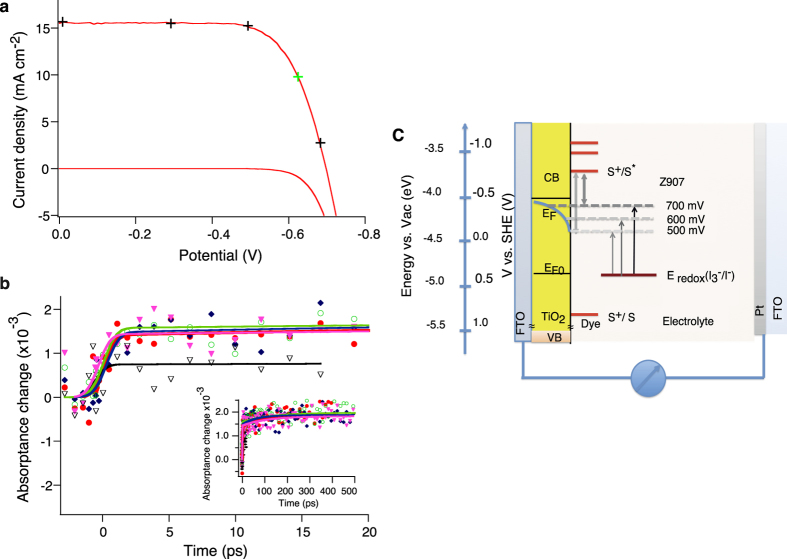
J-V analysis, pump-probe diffuse reflectance spectroscopy, and energy level diagram of the full device. (**a**) The J-V characteristics of an optimized standard DSC device based on Z907 dye and Z946 electrolyte measured in dark and under irradiance of AM 1.5G sunlight of 100 mW cm^−2^. (**b**) diffuse reflectance measurements on DSC under apply bias voltage. Signals are recorded at 840 nm reflecting the kinetic of oxidized dye molecules, Green circle: short circuit, Red circle: −500 mV, blue diamond: +500 mV, pink triangle: −320 mV, black triangle: −690 mV. (**c**) Energy level diagram of complete DSC. The energy level of quasi-Fermi level E_fn_ at the bias voltage of 300 mV and 700 mV are depicted. Trap state density exponentially increases with increasing the energy level. The E_fn_ level respect to E_f0_ at 3 different biases is illustrated. Trap density at voltage difference of 500 meV, 600 meV and 700 meV is approximately respectively 1 × 10^19^, 2 × 10^19^ and 7 × 10^19^ cm^−3^. Arrows show the shift in the quasi-Fermi level position and the energy difference of quasi-Fermi level and dye LUMO level.

We have combined the diffuse reflectance spectroscopy with potentiometric techniques to monitor the electron injection process in DSC standard device under working condition. Figure 5a shows the photovoltaic characteristics of the Z907 sensitizer and iodide based redox electrolyte device. The photovoltaic parameters of the device at full sunlight are; short circuit current density (Jsc) of 15.5 mA/cm^2^, open circuit photovoltage (Voc) of 698 mV, fill factor (FF) of 0.71 and power conversion efficiency (PCE) of 7.6%. Figure 5b presents the typical transient absorptance of the cell in short circuit condition and under different bias voltages of +500 mV, −500 mV (close to max power point) and −690 mV. Transient absorptance traces are not easily distinguishable. All traces in this figure can be fitted by exponential function with close fitting parameters; a component with a lifetime of 50–70 ps, and the flat behavior until hundred picoseconds, which is shown in inset. By raising the bias to −690 mV close to open circuit condition, the amplitude of the observed signal is almost half of the others while the kinetics remain similar. In other words, as the amplitude of the pump-probe signal is proportional to the number of oxidized dye molecules, with increasing the applied bias voltage the quantum yield of electron injection is reduced. The energy level diagram, which contains the energy level of the conduction band of TiO_2_ (CB), HOMO and LUMO level of Z907 dye and iodide-based redox electrolyte (Eredox) and trap state distribution are illustrated in Fig. 5c. Increasing the forward bias voltage would raise the quasi-Fermi level position by filling up the trap states to some extent in TiO_2_, which is highlighted in Fig. 5c. As it is observed by shifting the quasi-Fermi level toward the LUMO level of the dye, the energy difference gets noticeably smaller. In example the energy difference at the bias level of −600 mV is 75% of the energy difference at the bias level of −500 mV and further reduces to 50% at a higher bias level of −700 mV.

It should be noted that at the bias level of −690 mV, the amount of voltage in the films is less than this value due to the dark current. The voltage drop in the cell at a voltage bias of −700 mV is estimated as 90 mV. By taking account of this voltage drop, the respective amount of the cell photocurrent measured at bias voltages up to −500 mV and at −690 mV is consistent with the respective amplitude of the pump-probe signals. In our control studies (Supplementary Figures S6 and S7), the photovoltage induced by each laser pulse is estimated to be only of μV order when the cell is biased in the conventional voltages of hundreds of mV. This indicates that our pump-probe measurements under potentiostatic condition can be considered as a perturbation technique.

### Electron injection in DSC based on D-π-A organic sensitizer and cobalt electrolyte

**Figure 6:**
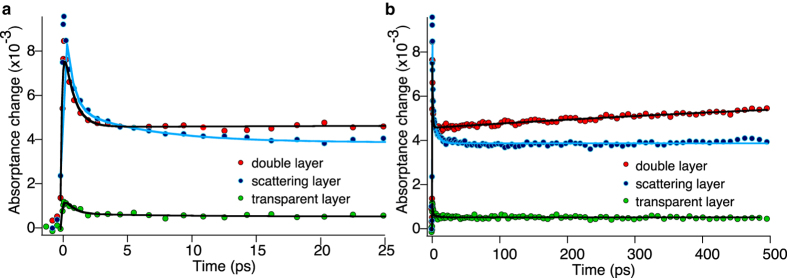
Femtosecond diffuse reflectance measurements on DSC devices based on organic D- π-A dye, Y123. Measurements are in the presence of cobalt-based redox mediator, Z1148 electrolyte. Red and blue markers are the measurements on the double layer and scattering layer films respectively and trace with green markers correspond to the transparent layer. Pump wavelength is 600 nm, and the probe wavelength is 840 nm. (**a**) The kinetics is drawn until 25 ps after excitation. In all three TiO_2_ films, the amplitude of the signal decays to half of its value in short time scale after excitation. (**b**) The kinetics are drawn until 500 ps after excitation. Solid lines are the result of fitting two exponential functions to the data.

One of the most significant advances in design of light-harvesting materials is the so-called donor-conjugated linker-acceptor (D-π-A) organic dyes. In comparison with Ru-based dyes, organic dyes have higher molar extinction coefficient and can be readily designed for a desired absorption spectrum^1^,^35^,^36^,^37^. These molecular structures look attractive in terms of electron donor-acceptor interactions^35^. In order to understand the electron injection dynamics in these systems, femtosecond diffuse reflectance spectroscopy is applied to DSC devices containing Y123 and a cobalt complex based redox electrolyte. Three different morphologies of the photoanode are used, and the results are compared in Fig. 6. In these measurements, the pump beam wavelength is 600 nm to excite the dye, and the probe beam is 840 nm. Upon laser excitation, an ultrafast formation of the signal happens in 200 fs, followed by a fast decay of the signal to 50% of its amplitude in 2 ps. After two picoseconds, the signal reaches a plateau in all the 3 morphologies of the TiO_2_ layers. Unlike Ru-based dyes, no obvious difference is observed in the kinetics for measurements in the presence of MPN solvent and redox electrolyte and the medium has no influence on the observed kinetics (measurements in the presence of MPN solvent are provided in Supplementary information Figure S9). The slow growth component of the signal in hundred picoseconds time scale seen in the films made of scattering particles or the double layer film, is assigned to the electron injection from dye aggregates. This component is removed from the signal when the sample is immersed in acetonitrile solvent for several hours (Supplementary Figure S10). TiO_2_ films prepared with scattering particles might have enough space to accommodate aggregated dye molecules within the pores, which are loosely in contact with the TiO_2_ layer. This makes a larger distance for electron transfer between the dye and TiO_2_.

**Figure 7:**
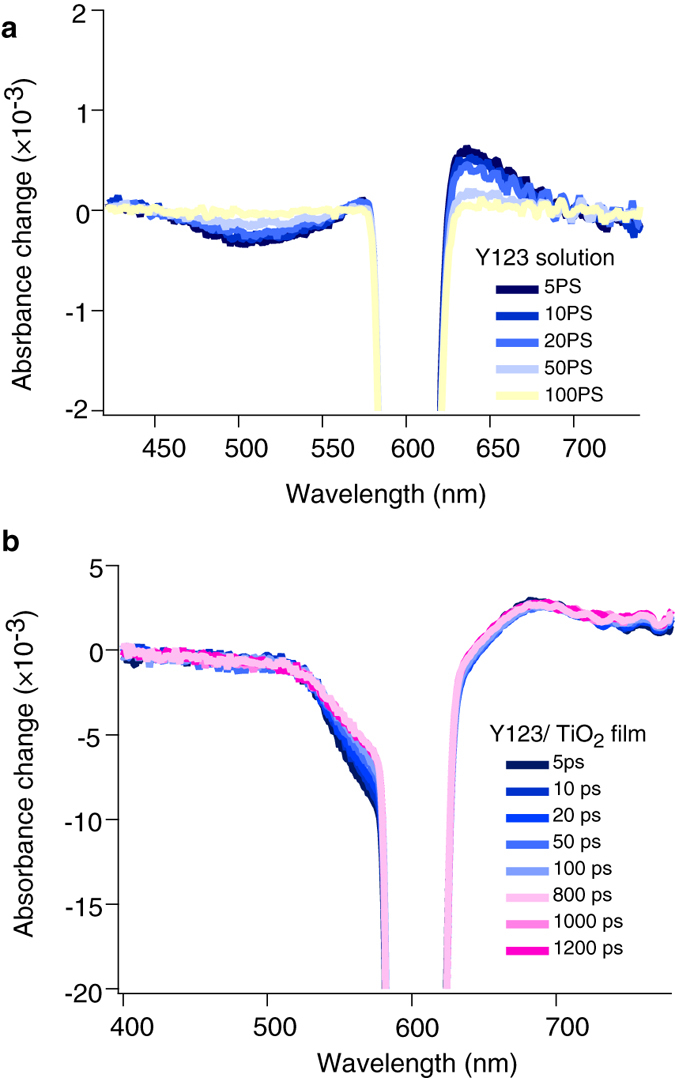
Transient white light continuum absorbance spectrum. (**a**) Y123 dye measured in solution and (**b**) Y123 sensitized TiO_2_ transparent film, in the visible light wavelength region. Excitation intensities are 300 nJ/ pulse for films and 1000 nJ/pulse for measurement in solution. Measurements in solution are performed under N_2_ bubbling.

In order to study the mechanism of the very fast decay of the signal in 2 ps, we performed the transient broadband absorption measurements. We compared the broadband transient absorption of Y123-sensitized TiO_2_ film with that of Y123 dye in solution as a reference sample where interfacial electron transfer process is deactivated. By comparison of the recorded spectra of the dye-sensitized films with the dye in solution we can clearly resolve the spectral absorption contribution of the excited-state and oxidized state of the Y123 dye. The transient absorption spectra of the samples measured at NIR region is also shown in Supplementary Figure S11. The ground state optical absorption spectrum of the dye is provided in Supplementary Figure S8. For the dye in solution (Fig. 7a), the negative peak at the characteristic ground state absorption of the dye around 520 nm, is attributed to the ground state bleaching of the dye formed upon photo-excitation. In Fig. 7a, a positive transient absorption is observed in the wavelength region from 630 nm up to 700 nm. This transition is assigned to excited-state absorption of dye molecule. The dye excited-state relaxation time constant is 52 ps and has a mirror-like kinetics to ground state relaxation. The transient absorbance spectrum of the dye-sensitized TiO_2_ films is presented in Fig. 7b. In this sample, the ground state bleaching is observed at 580 nm, which is around 60 nm shifted with respect to the steady-state absorption onset of the dye.

This red-shift in the transient absorption spectrum is an evidence of a Stark-shift of the absorption spectrum of the dye molecule. The Stark-shift is explained by the shift of ground state absorption of the dye molecule due to the local electric field induced by the electric dipole of the neighbor dye molecules. This effect was also previously reported for the same family of D-π-A dyes^36^,^37^. For the dye-sensitized TiO_2_ film the positive absorption feature is extended over the 630 nm wavelength regions. This positive feature is now assigned to a contribution of both of the excited-state absorption of the dye and the absorption by oxidized dye molecule formed upon injection of electrons into TiO_2_ conduction band.

**Figure 8:**
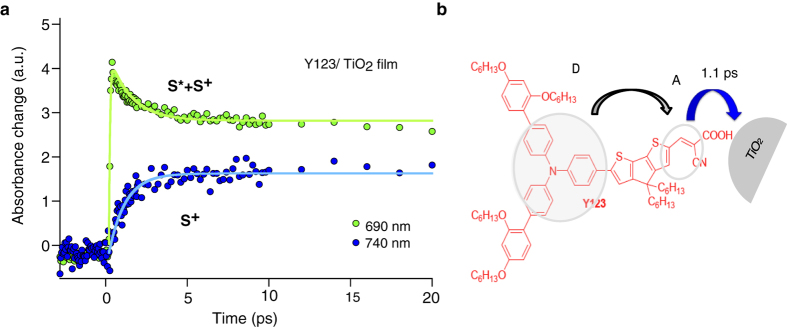
Comparison of the kinetics observed in the Y123 sensitized TiO_2_ film at different wavelength regions of 690 nm and 740 nm and Y123 molecular structure. (**a**) Transient absorptance of Y123 sensitized TiO_2_ films at 690 nm (green) and 740 nm (blue). The formation of the blue trace measured at 740 nm, which reflects the electron injection time, is occurring in picosecond time scale and is fitted with an exponential growth function with a time constant of 1.1 ps. (**b**) Y123 molecular structure and schematic of electron transfer processes. Blue arrow shows the electron injection process.

Figure 8 compares the kinetics of a Y123 sensitized TiO_2_ film probed in two different wavelength regions of 690 nm and 740 nm. According to Fig. 7a, at 690 nm the excited-state of Y123 has absorption while at 740 nm the excited-state does not absorb. Therefore, the blue trace in Fig. 8 recorded at 740 nm, represents a pure monitoring of the kinetics of the electron injection process. The formation of the signal, which reflects the electron injection time, is occurring in picosecond time scale and is fitted with an exponential growth function with a time constant of 1.1 ps. As a result, we observe that in the Y123 D-π-A dye, the charge injection kinetics is not as fast as in Ru-based dyes, as reported by Chergui and co-workers^28^.

The difference in the electron injection time in the Y123 with Ru-based dyes should be due to the D-π-A structure of this dye and its coupling with the TiO_2_ film. In the Y123 dye, the electron donor part is the triphenylamine unit, and the acceptor orbitals are located on the cyanoacrylate group. Cyclopentadithienophene (CPDT) is working as a π-bridge between the donor and acceptor parts for the conjugation of electrons. This bridge helps increasing the dipole moment and enhancement in the molar extinction coefficient of the dye molecule^38^. Our results suggest that the relaxation of the excited-state is fast with a time constant of 52 ps. This process competes with electron injection into the lower lying conduction band of TiO_2_. Moreover, non-adiabatic charge transfer from a molecular electronic excited state into a continuum of acceptor levels constituted by the conduction band of a semiconductor can be described by Fermi’s golden rule^2^. Due to the very high density of acceptor levels, the nuclear factor in the equation tends to a constant value. As a consequence, the thermodynamics of the process, the temperature, and the reorganization energy are not expected to affect the injection dynamics. The electronic coupling between the donor and the acceptor (electronic coupling matrix element squared |*H*]^2^) is then likely to control in a large extend the electron transfer rate. As |*H*|^2^ depends exponentially upon the charge transfer distance, the adsorption geometry and the electronic coupling between the dye’s HOMO and the empty *d*^*4*^ orbital manifold of Ti^IV^ sites on which the dye is anchored must be determining the electron injection time.

In summary, our experimental technique allowed us to reveal the charge separation dynamics in a complete opaque solar cell device under applied bias voltage. Also, the interfacial charge separation in different dye-sensitized opaque TiO_2_ nanostructured interfaces was determined. We showed that this technique could be a powerful and sensitive tool for measurements of opaque and highly absorbing materials. Coupling diffuse reflectance spectroscopy with potentiometric characterization tools gives a unique possibility to study charge carriers in devices under real operational conditions. In Ru-complex based solar cells, the kinetics of electron injection is confirmed to be ultrafast and is not affected by the bias voltage. In standard opaque and other TiO_2_ morphologies based devices, after ultrafast electron injection, an early recombination of photo-injected electrons with oxidized dye molecules is observed with features in the picosecond to nanosecond time scale. The respective amplitude of this recombination process is influenced by morphological parameter i.e. trap states in TiO_2_ films. We found that the ultrafast electron injection kinetics is not influenced by trap state filling upon increasing forward bias up to −500 mV. By applying a higher voltage close to open circuit conditions, and shifting the Fermi level of TiO_2_ closer to the dye excited-state level, the electron injection is less efficient but the injection kinetics is still ultrafast. For a DSC with a Y123 organic dye, the dynamic of the excited-state of the dye and the kinetics of electron injection process significantly differ from Ru-base dyes. The excited-state relaxation in the Y123 dye molecule competes with electron injection into TiO_2_. In contrast to Ru-complex based dyes, which show ultrafast electron injection in femtoseconds time scale, the electron injection for the Y123 dye is precisely monitored to occur within 2 ps after excitation of the dye.

Finally, the technique proposed here will be an excellent tool to be implemented for studies on highly absorbing materials, such as the new emerging perovskite based devices and can open up a new avenue of characterization research.

## Methods

### Pump-probe femtosecond diffuse reflectance spectrometer

In principle, the configuration of the diffuse reflectance spectroscopy is similar to the traditional transient absorption in transmission mode. The differences are: firstly in the probe beam geometry to collect the diffuse reflected light, which carries the information of transient species, secondly the sample structure and thirdly the optical model in data treatment. Here, a new optical scheme for collection of diffuse reflected light is designed which gives a unique time-resolution of sub-200 fs (Supplementary Figure S1). In this configuration, diffuse scattered light from the sample is collected, collimated, and focused onto the detector with two coupled off-axis 90° parabolic mirrors. Having this configuration, the large solid angle of light collection results in improved signal to noise ratio. The other advantage of using parabolic mirrors over lenses is that no further dispersion is introduced to the pulses; therefore, a better time-resolution is expected. Indeed, in this configuration, the time-resolution is limited by the time broadening of the beam in the diffusive sample. This time broadening is typically measured as about 30 fs in our samples^39^. For the transient absorption measurements, the pump beam at a defined wavelength is produced using a two-stage non-collinearly phase-matched optical parametric amplifier (NOPA). It is modulated using a synchronized chopper at a frequency of 0.5 kHz, which is half the repetition frequency of the laser. It is focused onto the sample at an angle of about 60° from normal. The pump beam has a diameter of 500 μm at the surface of the sample and typical energy of about 100–200 nJ/pulse. The probe beam is provided by a second NOPA having less energy than the pump on the sample to avoid multiple excitations and is focused having the spot size of around half of that of the pump. The polarization between the pump and probe beams is at the magic angle (54.7°). The transient response of the sample is measured by collecting the diffuse reflected pulses of the probe. The light scattered by the sample is focused onto the detector (photodiode: Nirvana detector, New Focus, model 2007). The signal of the detector is amplified by use of a lock-in amplifier. Lock-in parameters are set as integration time 1s, dwell time 4s, time constant 1s for measurements. A power supply (Weir) is used to apply a fixed bias voltage on the solar cell for diffuse reflectance measurements on the cell under voltage bias condition. The bias voltage between the two electrodes is changed from −690 mV to +500 mV and time-resolved diffuse reflectance of the device is measured at each applied bias. In these measurements, the pump and probe beam are irradiated to the cell from the backside (photoanode side), similar to the photovoltaic measurements. All the rest experimental details of experiments are similar to that previously explained.

### Time-resolution of diffuse reflectance setup and linearity tests

The time-resolution of the setup is defined by using optical Kerr gating technique. In this technique pump and probe beams are focused and spatially overlapped on a non-linear media (SF10 crystal or glass substrate). The cross-correlation of pump and probe beam on a Kerr-media is measured at an angle of 45° between the polarization of pump and probe. We performed the cross-correlation experiment in the diffuse reflectance mode. As the Kerr- media once the SF10 crystal and another time the scattering sample with cover glass is used. The reason is to compare the broadening of the cross-correlation peak when the diffuse reflectance is measured on these types of substrates. The time-resolution of the setup is sub- 200 fs. It should be noted that the time-resolution of this technique is limited by the time broadening of the beam in the sample due to the scattering effect. This time broadening is measured to be 120 fs for our samples. This is in contrast with the transmission based transient absorption technique in which the time-resolution is solely determined by the pulse duration of pump and probe and their cross-correlation. The setup has an unprecedented sensitivity as it enables measurements of transparent, non-reflective samples (see green curve in Fig. 6) with a reasonable signal to noise ratio. In order to check the linearity of measurements, the intensity of the pump beam is changed over a broad range of energy from 0.047 μJ/pulse to a high intensity of 0.950 μJ/pulse. The diffuse reflectance of dye-sensitized sample at a fixed time delay (i.e. 50 ps) is measured. Both absorptance and Kubelka-Munk formalism show a very good linear fit to the measurements over the whole intensities of excitations (Supplementary Figures S2 and S3).

### Data acquisition and treatment

In the configuration of reflectance measurements, the transient absorptance 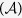 change can be measured and corrected by determining the absolute amount of diffuse reflected light with and without pump beam. This is practically achieved by chopping the pump pulse at half repetition frequency of the laser. The time-resolved diffuse reflectance of samples is measured by varying the delay time between the pump and probe pulses. Therefore, transient absorptance is displayed as:


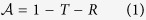


In case of opaque samples that the optical transmittance of sample is negligible, absorptance change (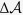) can be deduced only from reflectance change, as:


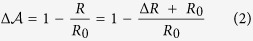


Where *T* is the intensity of the transmitted light and *R* and *R*_0_ represent the intensity of the diffuse reflectance of probe pulse with and without excitation, respectively. The linearity of absorptance change upon excitation intensity is tested in our control studies, over a wide range of excitation intensities.

Another theory describing the optical behavior for a tightly packed isotropic absorbing and scattering medium is Kubelka-Munk^40^,^41^, in which the Kubelka-Munk function relates the measurable so–called diffuse reflectance of the sample to the ratio of the absorption coefficient (K) and scattering coefficient (S). In the case of diluted medium, K is linearly dependent on concentration of absorbing species (c), in the same way, the Lambert-Beer Law is also valid in solutions; equation (3):


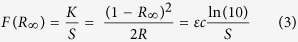


For quantitative simulation, use of the Kubelka-Munk function is essential, however, treating the transient reflectance of the samples with both equations of (2) and (3), does not change the kinetics.

### Broadband transient absorption setup

The pump−probe technique uses a compact CPA-2001, 1 kHz, Ti: Sapphire-amplified femtosecond laser (Clark-MXR), with a pulse width of about 120 fs at a wavelength of 775 nm. The pumping beam is generated using an NOPA tuned to 600 nm to generate pulses of approximately 8 μJ, that are then compressed in an SF10-glass prism pair down to duration of less than 60 fs (at FWHM). At the sample, the excitation pulse energy is decreased to a few hundred nJ. The probe beam is a white light continuum generated in a sapphire plate and splits before the sample into signals and reference beams in order to account for intensity fluctuations. Both beams were recorded shot by shot with a pair of 163 mm spectrographs (Andor Technology, SR163) equipped with 512 × 58 pixels back-thinned CCD cameras (Hamamatsu S07030-0906). The polarization of pump and probe pulses was set at a magic angle.

### Solar cell fabrication

Dye-sensitized solar cells were fabricated using a double-layered photoanode made of mesoporous TiO_2_ film. A transparent, 9 μm- thick layer of 20 nm particles was screen-printed onto an FTO glass plate (NSG-10, Nippon Sheet Glass). Subsequently, a 5 μm- thick layer of scattering particles (400 nm diameter) was deposited by screen-printing. The surface area of TiO_2_ film was 1 cm^2^. The TiO_2_ film was sintered up to 500 °C by a stepwise heating program. Prior and after TiO_2_ deposition a TiCl_4_ treatment was performed on the samples. The BET surface area of the mesoporous transparent film and scattering film were 85 m^2^ g^−1^ and 27 m^2^ g^−1^. The values of the two films porosity were 70% and 65% for transparent film and scattering film respectively. Prior to dye loading, photoanodes were sintered again at 480 °C for 30 minutes. Afterward, substrate was cooled down to 80 °C and immersed in the dye solutions for overnight. After rinsing with the acetonitrile, the stained substrates were sealed with pieces of thermally platinized electrode. The platinized electrode was made using a solution of H_2_PtCl_6_ on FTO glass (TEC15, Pilkington), and served as a counter electrode. The working and counter electrodes were separated by 25 μm-thick hot melt ring (Surlyn, DuPont) and sealed by heating. The electrolytes were introduced to the cells via pre-drilled holes in the counter electrodes.

### Photovoltaic characterization

The setup used for standard photovoltaic characterization (J–V curve) consisted of a 450 W Xenon lamp (Oriel), whose spectral output was matched in the region of 350–750 nm with the aid of a Schott K113 Tempax sunlight filter (Präzisions Glas & Optik GmbH), and a source meter (Keithley 2400) to apply potential bias and measure the photocurrent. A set of metal mesh filters was used to adjust the light intensity to a desired level. A black metal mask defined the cell active area to be 0.158 cm^2^.

### Preparation of dyes and electrolytes

Dyes and electrolyte composition are as following: Z907 dye solution: Z907 dye (0.3 mmol concentration) is dissolved in tert- butanol/ acetonitrile (ACN) solvent mixture (1:1 v/v). Y123 dye solution: Y123 dye dissolved in a 0.1-mmol dye solution in a tert- butanol/ acetonitrile mixture (1:1 v/v). Iodide based electrolyte coded Z946: I_3_^−^/I^−^ based electrolyte in 3-methoxypropionitrile (MPN) as solvent. 1.0 M DMII, 0.15 M I2, 0.5 M NBB, and 0.1 M GNCS in MPN. Cobalt based electrolyte coded Z1148: Co (II)/Co (III) based redox electrolyte in acetonitrile as solvent. 0.22M Co^II^(Bpy)_3_(B (CN)_4_)_2_,0.033M Co^III^(Bpy)_3_(B (CN)_4_)_3_, 0.1M LiClO_4_, 0.2M TBP in ACN.

## Additional Information

**How to cite this article**: Ghadiri, E. *et al.* Ultrafast charge separation dynamics in opaque, operational dye-sensitized solar cells revealed by femtosecond diffuse reflectance spectroscopy. *Sci. Rep.* **6**, 24465; doi: 10.1038/srep24465 (2016).

## Supplementary Material

Supplementary Information
