# Supplementary material for: Ultrafast charge separation dynamics in opaque, operational dye-sensitized solar cells revealed by femtosecond diffuse reflectance spectroscopy
Source: Sci Rep. 2016 Apr 20;6:24465. doi: 10.1038/srep24465 (PMC4837338; doi:10.1038/srep24465)
Supplement: Supplementary Information [file srep24465-s1.pdf]

# SUPPLEMENTARY INFORMATION

# Ultrafast charge separation dynamics in opaque, operational dye-sensitized solar cells revealed by femtosecond diffuse reflectance spectroscopy

Elham Ghadiri[1*], Shaik M. Zakeeruddin[2], Anders Hagfeldt[3], Michael Grätzel[2], and Jacques-E. Moser[1]

1 Photochemical Dynamics Group, 2 Laboratory for Photonics and Interfaces and 3 Laboratory of Photomolecular Science, Institute of Chemical Sciences and Engineering, Ecole Polytechnique Fédérale de Lausanne, CH-1015 Lausanne Switzerland

E-mail address: Elham.ghadiri@alumni.epfl.ch

## 1. Time-resolved diffuse reflectance spectroscopy

### State of the art pump-probe diffuse reflectance spectrometer

Figure S1 depicts the schematic of developed femtosecond time-resolved diffuse reflectance spectrometer. The new design of light collection configuration based on coupled off-axis parabolic mirrors allowed us to achieve a time resolution of sub-200 fs and collection of light with a big solid angle. The technique enables, the study of opaque solid films as well as highly absorbing systems.

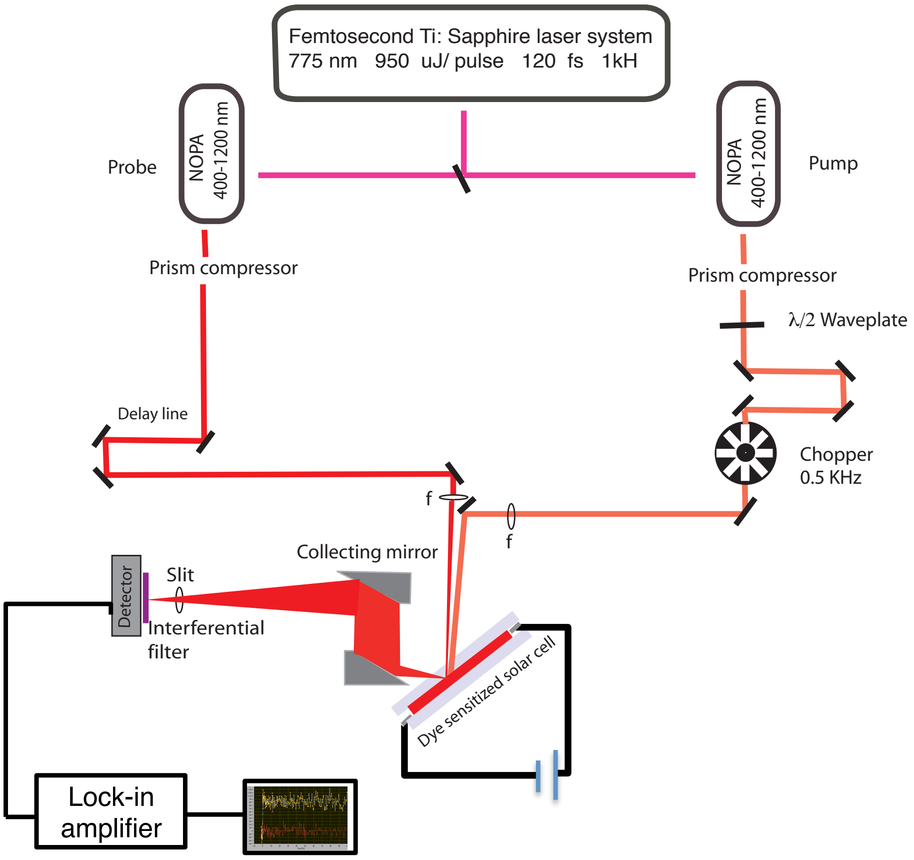


**Figure S1. Schematic of designed and developed femtosecond time-resolved diffuse reflectance spectrometer.** Setup is composed of a femtosecond laser, double stage non-collinear optical parametric amplifier (NOPA), parabolic light collecting mirrors, diode detector, and lock-in amplifier. The sample is a DSC device biased under working potential.

## Quantitative data analysis and optical modeling

Figure S2 shows the pump-probe diffuse reflectance signal at a delay time of 50 ps as a function of excitation intensity. The amplitude of the signal shows a good linear response to the excitation intensity tuned over a wide range.

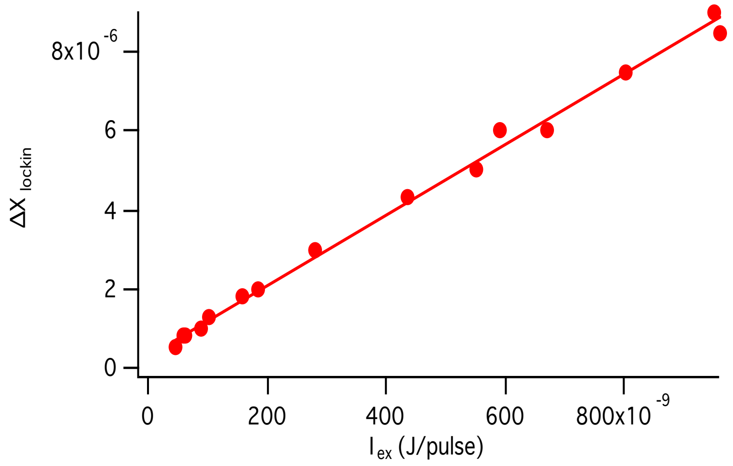


**Figure S2. Linearity test.** Transient diffuse reflectance measurements over a broad excitation pulse energy range are shown. The sample is a dye-sensitized double layer $TiO_2$ film. The pump beam energy is changed from 0.047 µJ to 0.95 µJ. The y-axis is the amplitude of the pump-probe signal at 50 ps after pulse excitation. The amplitude of the signal versus the excitation intensity shows a linear behavior.

The Kubelka-Munk formalism is applied to the time-resolved diffuse reflectance measurements according to equation (3) of the main text. The Kubelka-Munk function, which is representative of the concentration of absorbing species in the film, shows a perfect linear response over the excitation energy of pump beam.

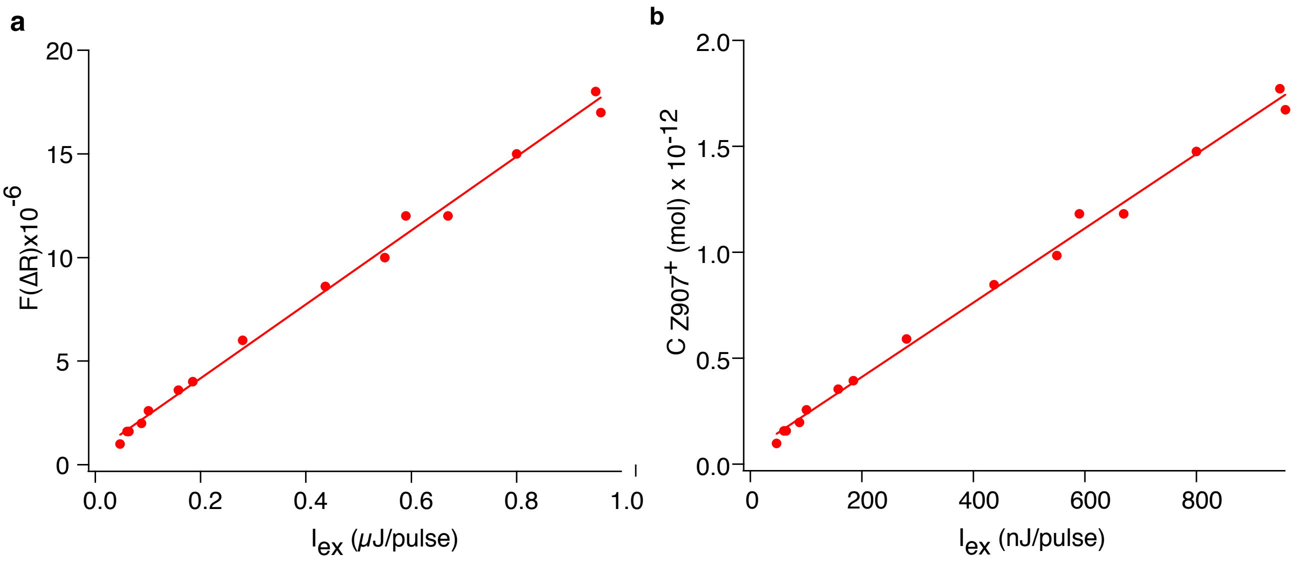


**Figure S3. Quantitative analysis of diffuse reflectance measurements.** The evolution of the transient Kubelka-Munk function upon an increase in excitation pulse energies are shown. The Kubelka- Munk function is integrated on the transient diffuse reflectance at a wavelength of 840 nm, measured on DSC based on Z907 sensitized double layer $TiO_2$ film. b) Extracted concentration of oxidized dye molecules at different excitation pulse energies deduced from Kubelka- Munk formalism. At a broad energy scale of pulse excitation, the obtained Kubelka-Munk function and consequently concentration of transient species show a linear trend.

## 2. Kinetics studies

### Influence of excitation intensity on the kinetics in Z907 sensitized complete photoanode

Intensity dependence of diffuse reflectance measurements on Z907 sensitized complete DSC photoanode (double layer) in the presence of MPN solvent is presented in Figure S4. The signals can be fitted with single exponential function. The observed kinetics at 840 nm is assigned to the early back recombination of photoinjected electrons with oxidized dye molecules. It should be noted that in kinetics studies all the measurements are performed at very low excitation intensities (below 300 nJ/ pulse).

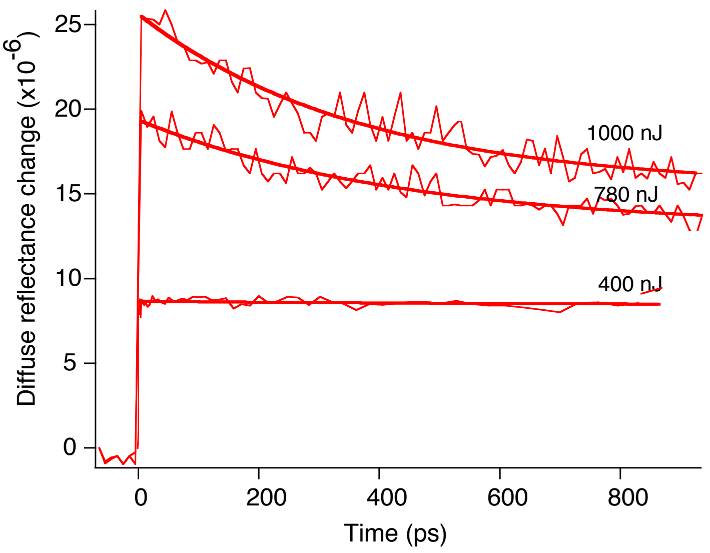


**Figure S4. Excitation intensity dependence test.** Transient diffuse reflectance measurements on Z907 sensitized $TiO_2$ double layer film immersed in MPN solvent at different excitation intensities. The probe beam wavelength is 840 nm. At the excitation of 1000 nJ, the kinetics is fitted with a double exponential. Excitation pulse energy is changed by one order of magnitude from 300 nJ to 1000 nJ. Consequently, the amplitude of the signal is raised, and the decay kinetics is accelerated. Rate constants of single exponential decay function fit to traces measured at 400 nJ, 780 nJ, and 1000 nJ are respectively, $0.0014 \times 10^{12}$ $s^{-1}$, $0.0023 \times 10^{12}$ $s^{-1}$ and $0.0028 \times 10^{12}$ $s^{-1}$. This depleting kinetics represents an early back reaction of electrons with oxidized molecules.

## Effect of probe wavelength

The evolution of oxidized dye molecule is also probed in the visible wavelength region at 670 nm and is compared with the measurements at 840 nm. The early back recombination kinetics is again observed when the diffuse reflectance measurements kinetics is recorded at 670 nm. The signal is fitted with single exponential function. The first order rate constant for measurement at 670 nm is $0.0008 \times 10^{12}$ $s^{-1}$ that corresponds to a time constant of 1.25 ns. Excitation intensity is 400 nJ/pulse.

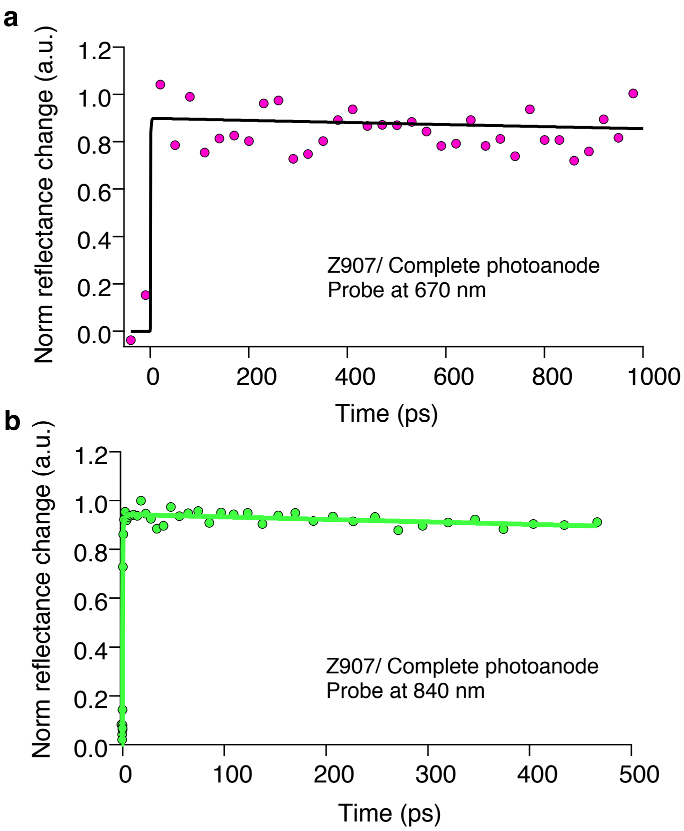


**Figure S5. Kinetics monitored at different probe wavelength.** Normalized diffuse reflectance change of Z907 sensitized double layer film recorded at 670 nm and 840 nm. The first order rate constant for measurement at 670 nm is $0.0008 \times 10^{12}\ s^{-1}$ that corresponds to a time constant of 1.25 ns. Excitation intensity is 400 nJ/pulse. The trace b is identical to the Figure 2c of the main text and is depicted here for comparison.

## Effect of $TiO_2$ film morphology and presence of electrolyte

To understand the effect of morphology on the kinetics of charge separation process, we also investigated the electron injection profiles in DSC devices based on different opaque nanostructured $TiO_2$ films like $TiO_2$ nanofibers, and $TiO_2$ nanotubes prepared by anodization of Ti foil. Figure 4-9 shows the dynamics of electron injection on Z907 dye-sensitized $TiO_2$ of various morphologies monitored at 840 nm. Panel a shows the kinetics of charge separation in Z907 dye-sensitized standard double layered based DSC device in the presence and absence of redox electrolyte. Similar measurement on Z907 dye-sensitized anodized $TiO_2$ nanotubes film in the presence of MPN solvent and redox electrolyte is also presented. It is interesting to observe that the electron injection is still in the ultrafast regime, and the recombination features are still present. It should be noted that the picosecond recombination feature is occurring in nanotubes with different relative amplitude compared to that of scattering particles.

Transient absorption studies on the band gap excitation of bare $TiO_2$ nanocrystalline films is performed by Furube and co-workers[1]. They have observed that after ultrafast formation of electron hole in $TiO_2$ particles, the surface trap electrons and surface trap holes forms in 200 fs (in the limit of time resolution) and relax to deep bulk traps in 500 ps. However, our measurements situation is slightly different with those studies in the sense that our samples are dye-sensitized $TiO_2$ films. In our system, the electrons are in the $TiO_2$ particles and holes are in the oxidized dye molecules. Although the photoinjected electrons can trap, in the same way, as what was observed for bare $TiO_2$ particles by groups of Colombo and Furube[1,2].

In anodized $TiO_2$ nanotube film, the trap states are energetically deeper than in the $TiO_2$ nanoparticle film as it was measured previously by modulated voltage-current techniques and terahertz spectroscopy[3,4]. So, the trap state distribution difference could play a vital role in decreasing the observed decreased relative amplitude of the picosecond recombination feature (i.e. the loss of electrons due to back recombination) in the study with nanotubes compared to nanoparticle film (black and green traces in 4a and 4b). Therefore, in nanotubes films, electrons that are trapped in the films are less free to undergo the early picosecond recombination in comparison to what is seen in particles.

**Modeling of the laser induced photovoltage in the solar cell and dark current correction**

In this model, a chemical capacitance is assigned to the DSC. The capacitance of the cell is measured by impedance spectroscopy, at each bias voltage. The chemical capacitance in the $TiO_2$ film increases (as the applied forward bias is increased). The amount of photoinjected charge into this capacitor is estimated based on quantification of the transient diffuse reflectance spectrum. Kubelka-Munk formalism is integrated on the diffuse reflectance spectrum, as it is proportional to the concentration of absorbing species.

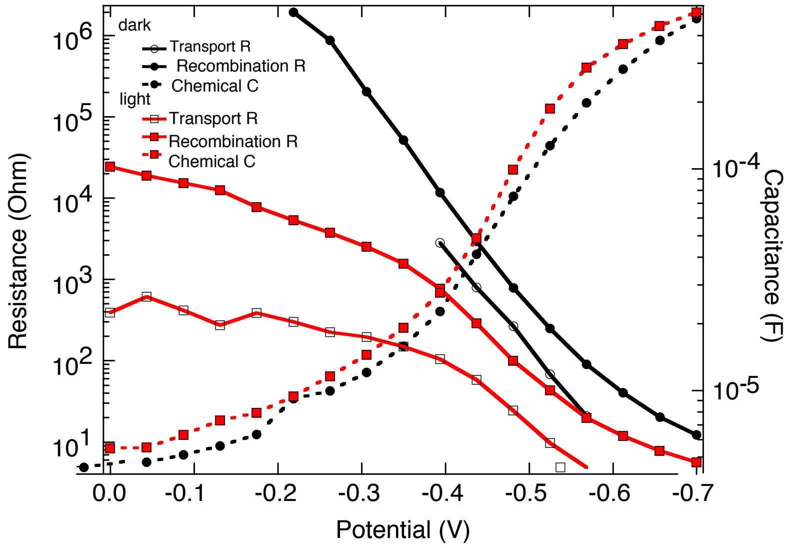


**Figure S6. Impedance spectroscopy.** Impedance measurements on DSC based on Z907 dye and Z946 electrolyte. The cell active area is 0.283 $cm^2$.

Having:

$$\Delta Q = C\Delta V \qquad \text{(S1)}$$

$$\Delta Q_{Pulse} \cong C^{+}_{Z907} \qquad \text{(S2)}$$

The photovoltage induced by each laser pulse is estimated to be about 10 μV at 700 mV bias voltage and 38 μV when the cell is biased at 520 mV. However for laser spectroscopy measurements on the cell at short circuit condition or small bias voltage, the amount of laser induce shift in the quasi-Fermi level position is in the order of some mV.

A good approximation of dark current correction is to consider the cell series resistance and according to Ohm law draw the voltage drop in the cell due to dark current.

$$\Delta V_{Voltage\ drop} \cong R_{Series} I_{dark} \qquad \text{(S3)}$$

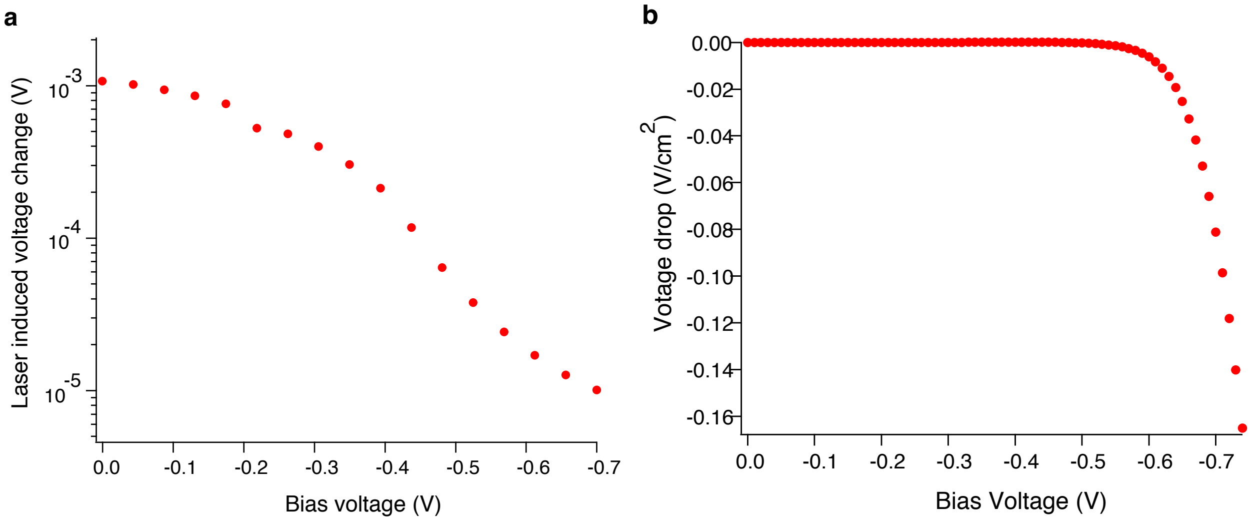


**Figure S7. Laser induced voltage rise and voltage drop due to dark current in DSC.** a) The amount of laser pulse induces change in the voltage of the solar cell when the pump-probe experiments are performed on the solar cell under operational condition is calculated. The amount of laser induced voltage raise compared to the applied bias voltage is negligible. b) The voltage drop at the cell filled by active layer mainly due to dark current at different bias voltages. The voltage drop is estimated from Ohm law and the measured series resistance in the solar cell device. The I-V curve of the device is shown in figure 5a of the main text.

## Comparison of the photovoltaic and optical response of two different DSC devices made based on transparent layer, and scattering layer film of same thickness

Two types of DSC devices based on two different $TiO_2$ films with same thickness sensitized with Z907 dye were made. The first device is based on a 5 µm-thick $TiO_2$ film made of scattering particles and the second device is made of a 5 µm-thick transparent $TiO_2$ layer. All preparation and test situation were identical for both devices. The morphological parameters, optical properties and photovoltaic performances of the devices are depicted in Table S1.

As depicted in Table S1, in the scattering $TiO_2$ layer, the BET surface area is 27 $m^2$/gr and the roughness factor of the film is 36.4 /$cm^2$ µm. These values are much smaller than those in transparent layer film being 85 $m^2$/gr and 98 /$cm^2$ µm respectively. Therefore a smaller amount of dye is adsorbed on the scattering layer film. Therefore, the value of the maximum light absorptance ($\mathcal{A}$) in the scattering layer is 0.66 and is smaller than that in the transparent film being 0.88. The photocurrent generated in a DSC device made of transparent layer is 11.4 mA/$cm^2$ while this value for scattering $TiO_2$ layer based device is only 5.9 mA/$cm^2$. The difference in the photocurrent is due to the amount of adsorbed dye molecule in the two films. For a fair comparison, the photocurrent of

devices is normalized to the absorptance of the film. The obtained value for the transparent layer is 12.95 while for scattering layer is only 8.93. Therefore, the amount of J/ $\mathcal{A}$ (current normalized to the absorptance) for the scattering film is 30% less than that of the transparent layer. This observation indicates that in the scattering layer based device about 30% of the photo-generated electrons are lost. This can be in agreement with our laser spectroscopy results that for big particles we witnessed loss of electrons due to prompt back recombination.

**Table S1. Comparison of the structural, optical and photovoltaic properties of two DSC devices based on scattering layer and transparent layer of identical thickness.**

| $TiO_2$ film | Film roughness factor ($cm^{-2}$ $\mu m^{-1}$) | BET Surface area ($m^2$/gr) | Absorptance ($\mathcal{A}$) | Jsc (mA) | Jcs/ $\mathcal{A}$ (mA) | Voc (mV) |
|---|---|---|---|---|---|---|
| Scattering layer (5 μm) | 36.4 | 27 | 0.66 | 5.9 | 8.93 | 704.9 |
| Transparent layer (5 μm) | 98 | 85 | 0.88 | 11.4 | 12.95 | 697.6 |

## 3. Electron injection in Y123 dye-sensitized solar cell devices

Figure S8 illustrates the steady-state absorbance spectrum of Y123 dye measured in solution. The first absorption peak of the dye is at 530 spectral regions.

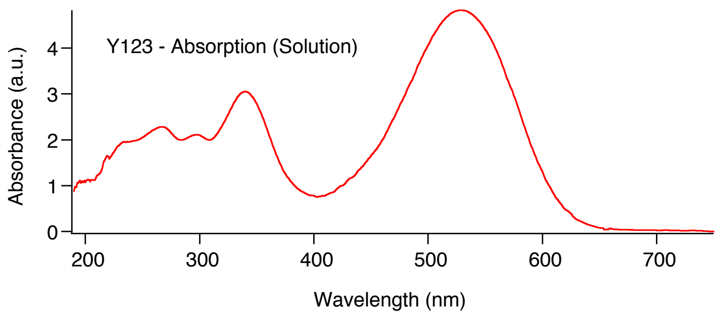


**Figure S8. Optical analysis.** Steady-state absorbance spectrum of the Y123 dye in solution.

## Effect of liquid environment

Figure S9 depicts the measurements on Y123 dye-sensitized different $TiO_2$ films in the presence of only MPN solvent probed at 840 nm. Comparing with the measurements in the presence of the electrolyte, which is shown in figure 6 of the main text, no obvious difference is observed, and the kinetics are independent of the environment.

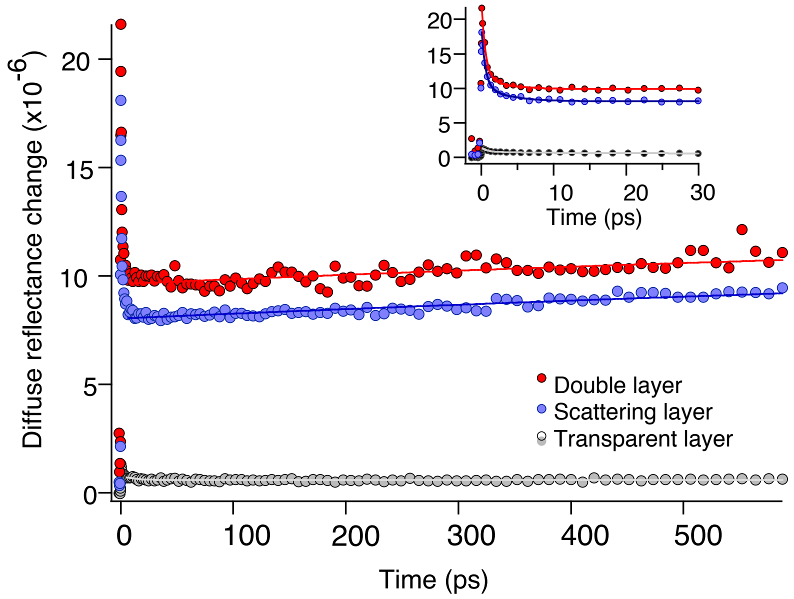


**Figure S9. Femtosecond diffuse reflectance measurements on Y123 dye-sensitized $TiO_2$ films in the presence of MPN solvent.** Red markers (double layer), blue markers (scattering layer) and gray markers (transparent layer). The time constants of the exponential fit to the decay component of the signals are: $\tau_1$= 7.249 ps and $\tau_2$= 0.663 ps for the transparent film, $\tau_1$= 3.623 ps and $\tau_2$= 0.552 ps for the scattering film and $\tau_1$= 3.116 ps and $\tau_2$= 0.553 ps for the double layer film.

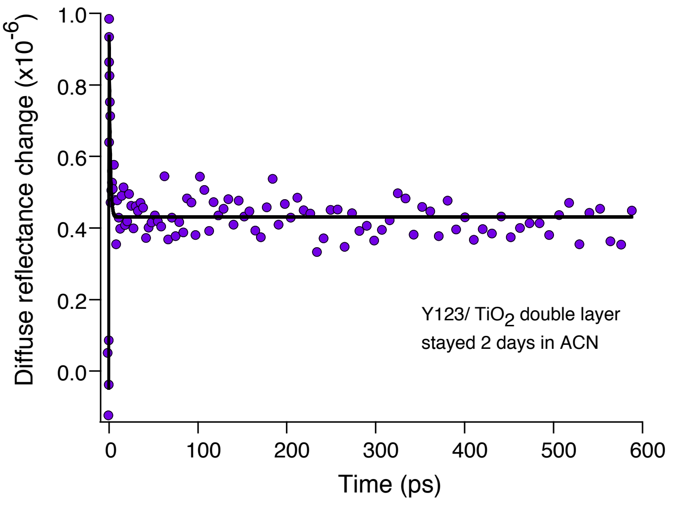


**Figure S10. Effect of acetonitrile treatment.** Femtosecond diffuse reflectance measurement on organic Y123 dye-sensitized $TiO_2$ double layer film immersed two days in acetonitrile after dye loading.

Figure S10 shows the measurements on double layer film immersed for two days in acetonitrile, which is the same sample for which the kinetics is shown in the red trace in figure 6 of the main text. We observe that after immersing in acetonitrile, the slow rise kinetics is removed, and a flat kinetics is replaced. This is due to dissolving of the extra dye molecules attached to the surface of the film in the form of dye aggregates.

Figure S11a shows the transient absorbance spectrum of the Y123 dye in solution. The pump excitation wavelength in these measurements is 600 nm. A negative peak around 520 nm is observed which can be assigned to the bleaching of the ground state absorption of the dye upon photo-excitation. This peak is consistent with the optical absorption peak of the dye in figure S8. The recovery of the ground state bleaching of the dye is fast. This kinetics can be fitted with a single exponential function indicating a time constant of 57 picoseconds. A positive peak is observed in the wavelength region from 630 nm to 680 nm. The small positive feature in the spectrum around 565 nm can be a tail of the same absorption feature, which is partially, covered by laser pulse excitation at 600 nm. The positive peak can be assigned to the excited state absorption of the Y123 dye. This deactivation of the Y123 dye excited state can be fitted to a single exponential function with almost the same lifetime of about 50 ps and, therefore, has a mirror-like kinetics to ground state bleaching. A small negative peak at the NIR region about 750 nm is assigned to the emission of the dye.

Figure S11b shows the spectrum of the transient absorbance change of the dye anchored on $TiO_2$ films. It is observed that negative peak at 500 nm that is assigned to the ground state bleaching, when the dye is anchored onto $TiO_2$ surface, is very small and a clear red-shift in the bleaching of the ground state absorption of about 60 nm is observed. Although this red-shifted pick is overlapped and partially covered with the excitation beam at 600 nm, it is clearly resolved. This red- shift in the transient absorption spectrum of the dye molecule is probably an evidence for Stark-shift of the ground state absorption of the dye molecule. The Stark-shift effect was previously observed in other organic dyes by Boschloo et al. and in Ru-based dyes by Meyer and co-workers. This observation is explained by the shift of ground state absorption of the dye molecule, which is influenced, from the

local electric field induced by the electric dipole of the neighbor dye molecules. Finally, the positive feature above 630 nm in solid sample is now assigned to a contribution of both of the excited state absorption of the dye and the absorption by oxidized dye molecule formed upon injection of electrons into $TiO_2$ conduction band.

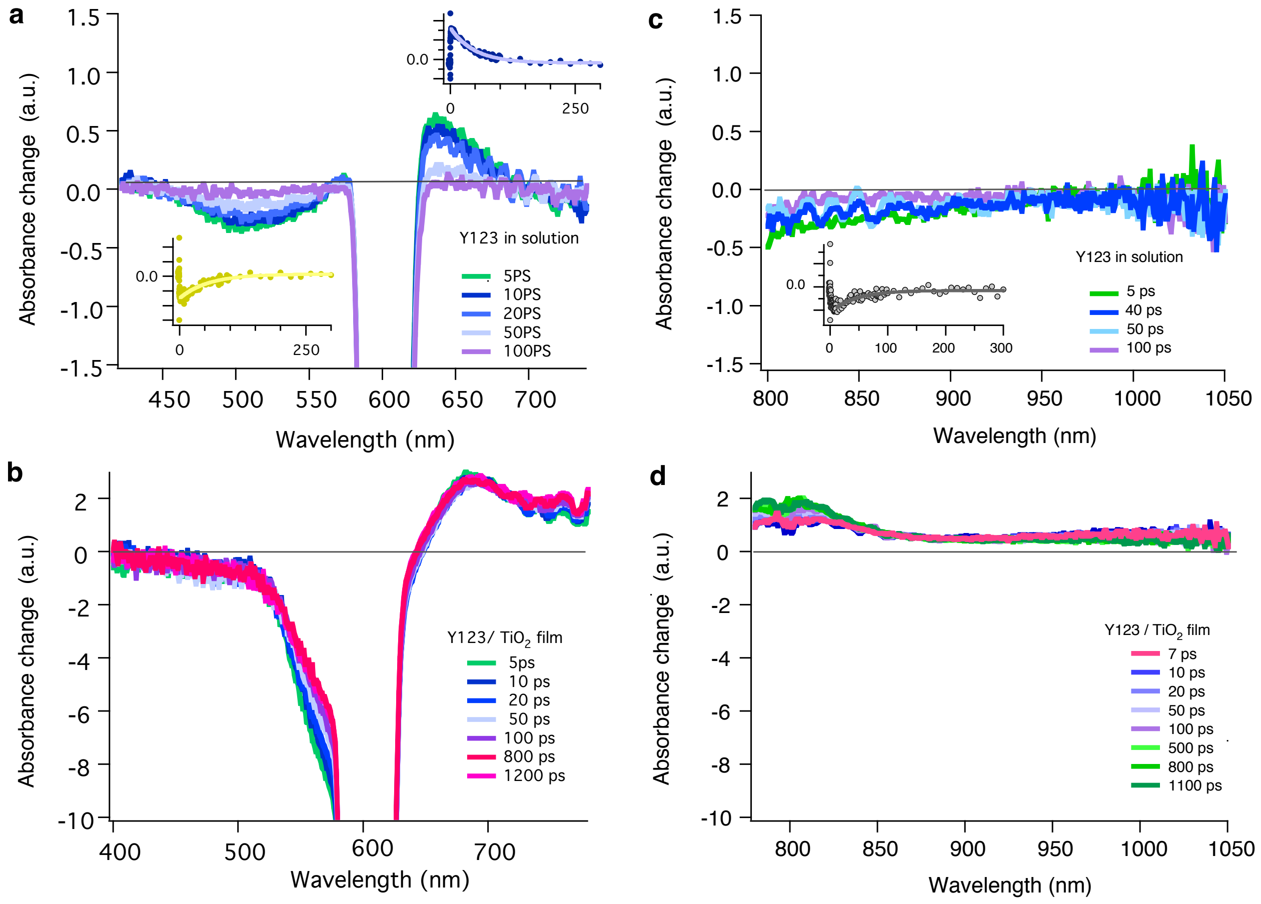


**Figure S11. Transient white light continuum spectrum.** a) Y123 dye measured in solution and b) Y123 sensitized $TiO_2$ transparent film, in the visible light wavelength region. The pump excitation is at wavelength 600 nm. c,d) NIR transient white light continuum spectrum of c) Y123 dye measured in solution and d) Y123 sensitized $TiO_2$ transparent film, in NIR wavelength region. The excitation wavelength is at wavelength 600 nm. Excitation intensities are 300 nJ/ pulse for films and 1000 nJ/ pulse for measurement in solution.

Figure S11c and S11d depict the NIR transient absorbance spectrum of the Y123 dye in solution and anchored on $TiO_2$ film. The excitation wavelength at these measurements is 600 nm. In the transient spectrum of the dye in solution, the negative peak at NIR region up to 950 nm can be due to the emission of the dye. The spectrum of the Y123 sensitized $TiO_2$ film shows a contribution of both absorption of oxidized dye molecules and dye emission.

**References:**


1. Tamaki, Y. et al. Dynamics of efficient electron-hole separation in $TiO_2$ nanoparticles revealed by femtosecond transient absorption spectroscopy under the weak-excitation condition. *Phys. Chem. Chem. Phys.* **9**, 1453 (2007).
2. Colombo, D. P. & Bowman, R. M. Femtosecond diffuse reflectance spectroscopy of $TiO_2$ powders. *J. Phys. Chem.* **99**, 11752–11756 (1995).
3. Mohammadpour, R., Iraji zad, A., Hagfeldt, A. & Boschloo, G. Comparison of Trap-state Distribution and Carrier Transport in Nanotubular and Nanoparticulate $TiO_2$ Electrodes for Dye-Sensitized Solar Cells. *Chemphyschem* **11**, 2140–2145 (2010).
4. Richter, C. & Schmuttenmaer, C. A. Exciton-like trap states limit electron mobility in $TiO_2$ nanotubes. *Nat Nanotechnol* **5**, 769–772 (2010).
